# Serum ferritin levels are positively associated with the severity of schizophrenia symptoms

**DOI:** 10.1192/j.eurpsy.2026.12127

**Published:** 2026-07-31

**Authors:** A. Turek, N. Śmierciak, M. Szwajca, W. Krzyściak, P. Donicz, K. Furman, M. Pilecki

**Affiliations:** 1 Department of Child and Adolescent Psychiatry and Psychotherapy, Chair of Psychiatry, Faculty of Medicine; 2 Department of Medical Diagnostics, Jagiellonian University Medical College, Krakow, Poland

## Abstract

**Introduction:**

Research studies have shown abnormal iron metabolism parameters, including altered iron, ferroportin, and ferritin concentrations, in schizophrenia. In recent years, the issue has been re-examined using increasingly modern methods, where precise post-mortem and imaging studies show firm results indicating abnormal iron metabolism in the brains of people with schizophrenia. Serum ferritin levels could be associated with abnormal iron metabolism in the central nervous system in schizophrenia. Alternatively, ferritin as an acute-phase protein may be involved in immune dysregulation in schizophrenia. Altered brain iron metabolism of patients with schizophrenia may influence neuronal functionality and potentially be reflected through the clinical manifestation of schizophrenia symptoms.

**Objectives:**

To assess the relationship between serum ferritin concentration and the clinical state of schizophrenia. Additionally, to assess differences in serum ferritin concentration between people with schizophrenia and healthy controls.

**Methods:**

36 adult patients (33.3% female) with schizophrenia in remission (SCZ) and 38 adult healthy controls (CON) (59.7% female), matched with age, had their blood taken in the morning, before the first meal and medication. A set of blood tests, including blood ferritin concentration, was performed at the laboratory of the University Hospital in Krakow. Patients with schizophrenia underwent a precise psychiatric interview, using the Positive and Negative Syndrome Scale (PANSS), Scale for the Assessment of Negative and Positive Symptoms (SANS, SAPS). The Mann-Whitney U-test was used for intergroup analysis. Spearman’s rank correlation coefficient analysis was performed to test associations between parameters in separate groups.

**Results:**

Groups did not differ significantly in serum ferritin concentration (SCZ: Me=83.5 ug/L (Q25-75=53.5-147.75); CON: Me=65.5 ug/L (Q25-75=28-105); p=0.16). In the SCZ, serum ferritin levels correlated significantly, and positively with PANSS total score (r=0.47;p<0.01), SANS score (r=0.49;p<0.01) SAPS score (r=0.37;p<0.05) and following sums of PANSS subscales: Negative scale (r=0.47;p<0.01), Positive scale (r=0.39;p<0.05), General Psychopathology (r=0.44;p<0.01), Negative symptoms (r=0.52;p=0.001), Positive symptoms (r=0.41;p<0.05), Uncontrolled Hostility/Excitement (r=0.34;p<0.05), Anxiety/Depression (r=0.38;p<0.05). Serum ferritin levels did not correlate significantly with the age of disease onset, treatment duration, neuroleptic dose equivalent, or the number and frequency of hospitalisations.

**Conclusions:**

Patients with schizophrenia do not show significantly altered serum ferritin levels compared to healthy controls. The severity of schizophrenia symptoms is positively associated with serum ferritin levels. Further investigation of iron metabolism in schizophrenia may develop new insights into the nature of the clinical presentation of schizophrenia.

**Disclosure of Interest:**

None Declared

